# Supplementary material for: Segregated basal ganglia output pathways correspond to genetically divergent neuronal subclasses
Source: bioRxiv. 2024 Sep 18:2024.08.28.610136. Originally published 2024 Aug 29. Preprint. [Version 2] doi: 10.1101/2024.08.28.610136 (PMC11383992; doi:10.1101/2024.08.28.610136)
Supplement: 1 [file NIHPP2024.08.28.610136v2-supplement-1.pdf]

bioRxiv preprint doi: https://doi.org/10.1101/2024.08.28.610136; this version posted September 18, 2024. The copyright holder for this preprint (which was not certified by peer review) is the author/funder, who has granted bioRxiv a license to display the preprint in perpetuity. It is made available under aCC-BY 4.0 International license.

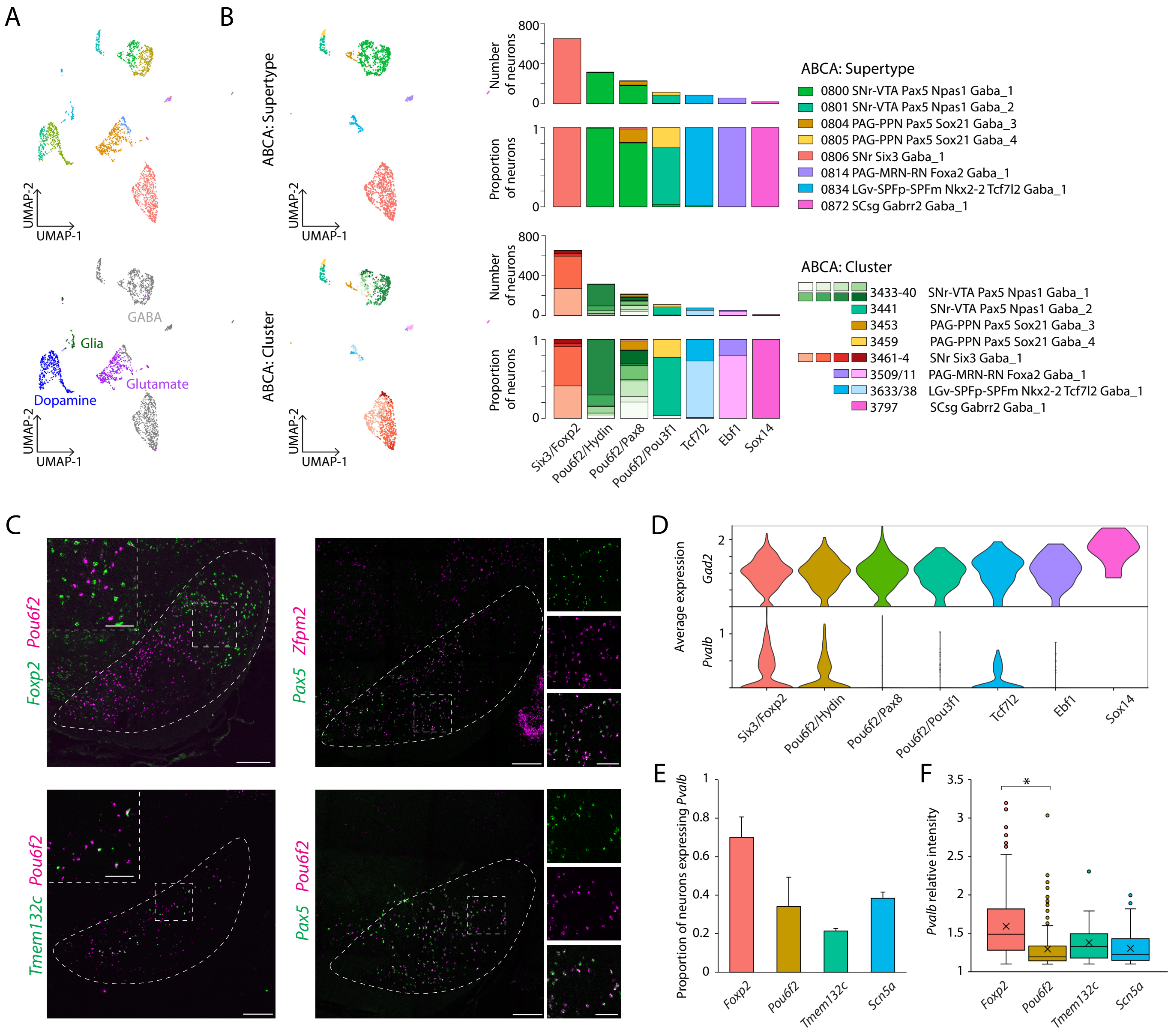

Figure S1: Characterization of SNr cell types, related to Figure 1

(A) UMAP representation of 2596 nuclei isolated after SNr microdissection, colored by assigned cluster (top) and cell type label (bottom).

(B) SNr cluster identity mapped to Allen Brain Cell Atlas mouse taxonomy with MapMyCells. UMAP representations of SNr GABAergic neurons from 1B colored by ABCA supertype (top left) or ABCA cluster (bottom left). Number and proportion of neurons per cluster mapping to corresponding ABCA label (right).

(C) RNAscope reveals marker distribution within SNr. Scale bars are 250µm, inset and rightside panel scalebars are 100µm.

(D) Violin plots showing average expression of *Gad2* and *Pvalb* per SNr cluster.

(E) Proportion of *Foxp2*$^{+}$, *Pou6f2*$^{+}$, *Tmem132c*$^{+}$ and *Scn5a*$^{+}$ neurons labeled by RNAscope that colocalize with *Pvalb*. N = 3 mice, 6 slides, *Foxp2*: 279 neurons, *Pou6f2*: 335 neurons, *Tmem132c*: 150 neurons, *Scn5a*: 187 neurons.

(F) Relative intensity of *Pvalb* expression in *Foxp2*$^{+}$, *Pou6f2*$^{+}$, *Tmem132c*$^{+}$ and *Scn5a*$^{+}$ neurons colocalizing with *Pvalb*. N = 3 mice, 6 slides, *Foxp2*: 225 neurons, *Pou6f2*: 153 neurons, *Tmem132c*: 31 neurons, *Scn5a*: 71 neurons. Data are represented as mean ± SEM. *: $p < 0.0001$.

bioRxiv preprint doi: https://doi.org/10.1101/2024.08.28.610136; this version posted September 18, 2024. The copyright holder for this preprint (which was not certified by peer review) is the author/funder, who has granted bioRxiv a license to display the preprint in perpetuity. It is made available under aCC-BY 4.0 International license.

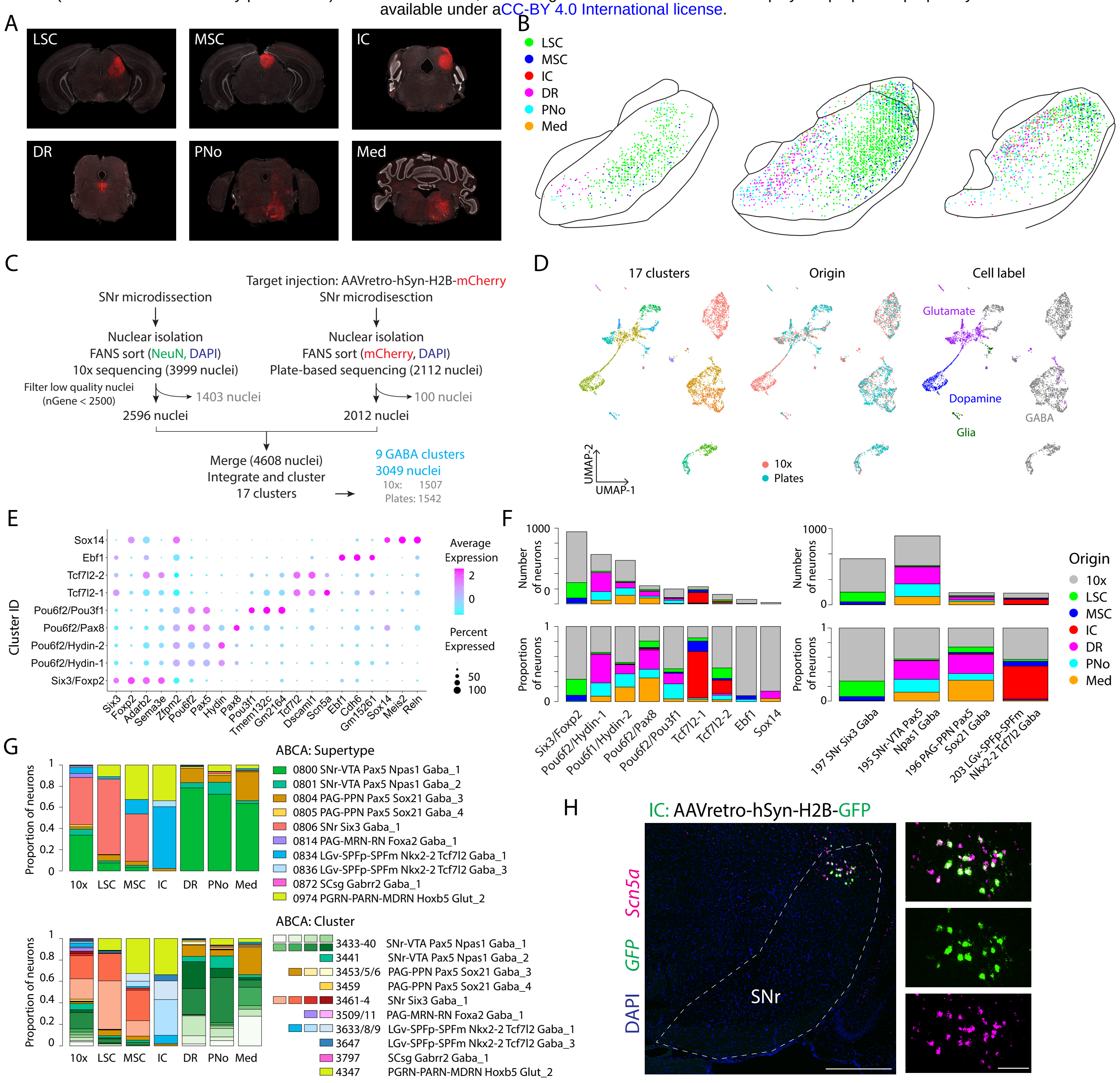

Figure S2. Characterization of projection-defined SNr cell types, related to Figure 2
(A) Six SNr target sites injected with AAVretro-hSyn-H2B-mCherry.
(B) Reconstruction of coordinate locations of SNr neurons retrogradely labeled by injection of AAVretro-hSyn-H2B-mCherry in each of six target sites.
(C) Experimental schematic for integrated analysis of 10x SNr profiling and single-nucleus RNAseq profiling of retrogradely labeled SNr neurons.
(D) UMAP representation of 4608 SNr neurons from integrated cluster analysis of whole SNr 10x (2596 neurons) and projection-tagged plate-based RNAseq (2012 neurons), colored by assigned cluster, origin or cell type label.
(E) Dotplot showing expression of selected marker genes across clusters.
(F) Stacked barplot showing the number and proportion of SNr neurons per cluster (left) and mapped ABCA subclass (right) originating from the 10x dataset or each projection target.
(G) Proportion of 10x or retrogradely labeled SNr neurons corresponding to a given ABCA supertype (top) or ABCA cluster (bottom).
(H) SNr neurons retrogradely labeled by IC injection of AAVretro-hSyn-H2B-GFP colocalize with *Scn5a*. Scalebars are 500µm (left) and 100µm (right panels).

bioRxiv preprint doi: https://doi.org/10.1101/2024.08.28.610136; this version posted September 18, 2024. The copyright holder for this preprint (which was not certified by peer review) is the author/funder, who has granted bioRxiv a license to display the preprint in perpetuity. It is made available under aCC-BY 4.0 International license.

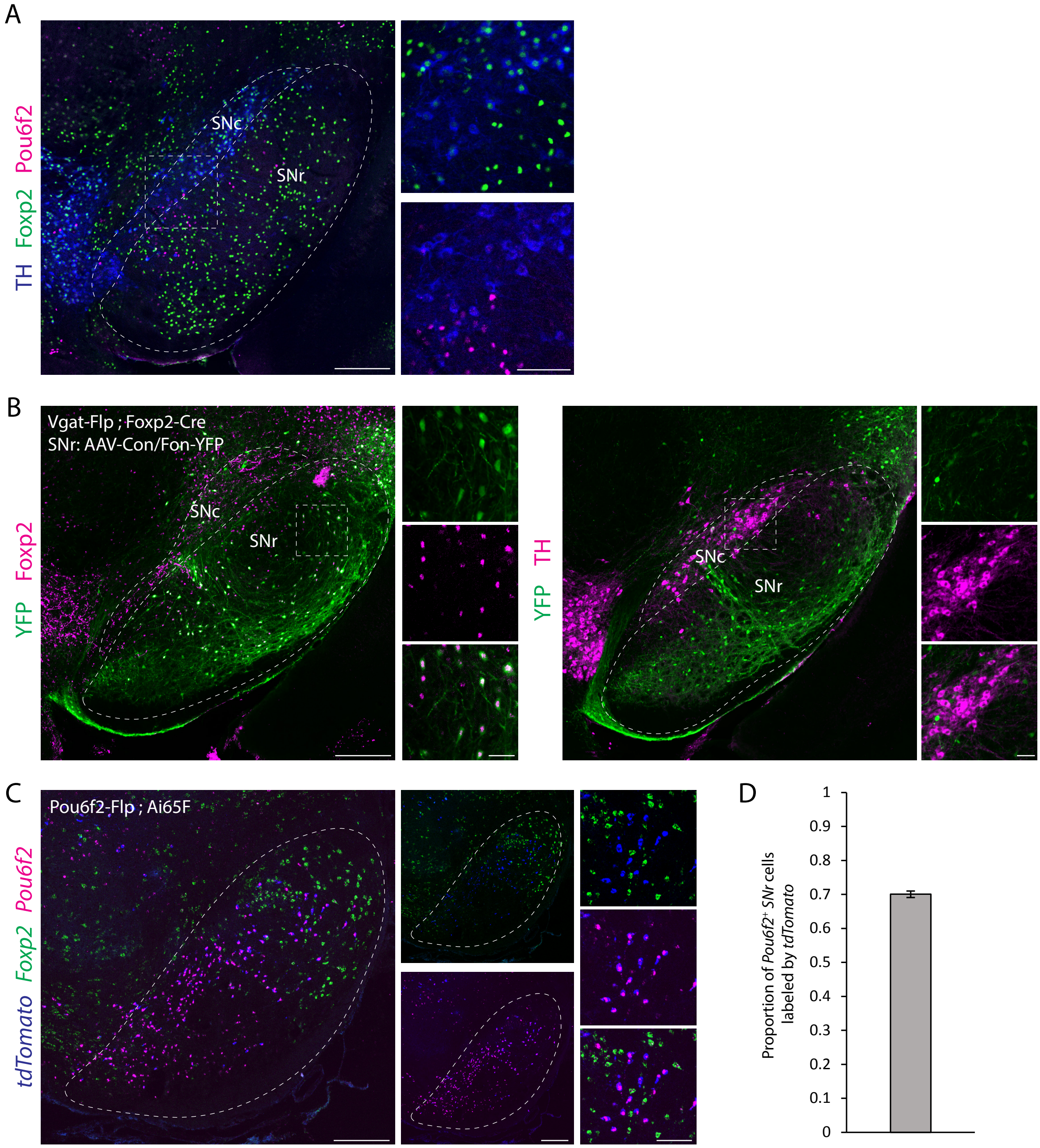

Figure S3. Validation of Foxp2 and Pou6f2 subclass labeling strategies, related to Figure 3

(A) TH⁺ dopamine neurons in the substantia nigra pars compacta (SNc) express Foxp2. SNc adjacent Pou6f2⁺ SNr neurons do not express TH. Scalebar is 250µm (left) and 100µm (right)

(B) Injection of AAV-Con/Fon-YFP into the SNr of Vgat-Flp;Foxp2-Cre mice labels TH⁻/Foxp2⁺ cells in SNr but not TH⁺ cells in SNc. Scalebar is 250µm (large panels) and 50µm (small panels).

(C) Validation of Pou6f2-Flp mouse line with RNAscope. *TdTomato*⁺ cells in Pou6f2-Flp;Ai65F mice colocalize with endogenous expression of *Pou6f2* but not *Foxp2*. Scalebars are 250µm (left and middle panels) and 100µm (right panels).

(D) Proportion of SNr neurons expressing endogenous *Pou6f2* by RNAscope that are labeled by *tdTomato* in Pou6f2-Flp;Ai65 mice. N = 3 mice, 6 sections, 3870 *Pou6f2*⁺ neurons. Data are represented as mean ± SEM.

bioRxiv preprint doi: https://doi.org/10.1101/2024.08.28.610136; this version posted September 18, 2024. The copyright holder for this preprint (which was not certified by peer review) is the author/funder, who has granted bioRxiv a license to display the preprint in perpetuity. It is made available under aCC-BY 4.0 International license.

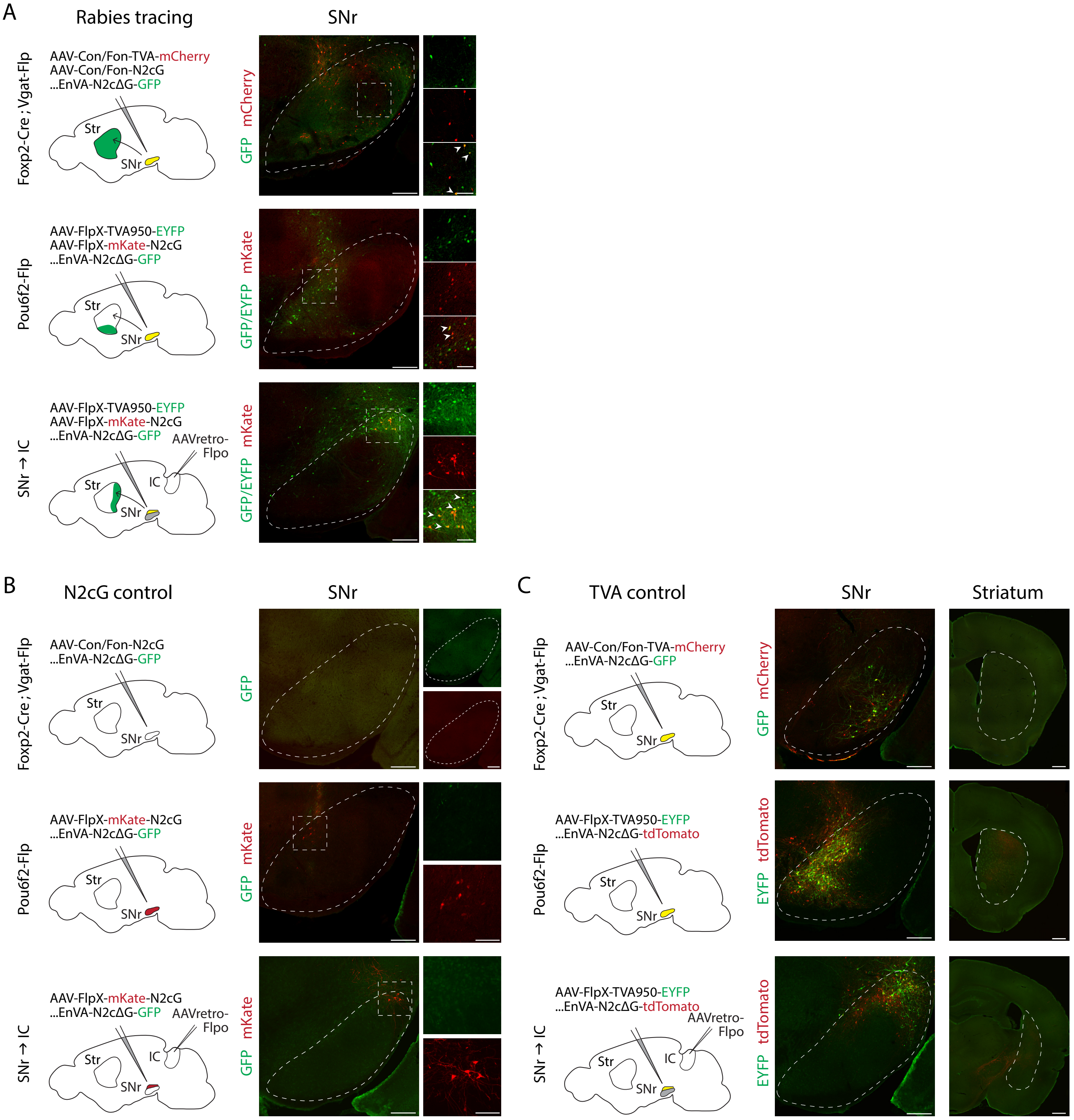

Figure S4. Validation of rabies labeling strategy, related to Figure 4

(A) Starter cells in SNr labeled with rabies virus after injection of TVA and N2cG helper viruses. Scalebars are 250µm (left) and 100µm (right).

(B) Rabies virus does not label cells in SNr after injection of N2cG helper virus without TVA helper virus. Scalebars are 250µm (left panels and top right small panels) and 100µm (middle and bottom small panels).

(C) Rabies virus labels cells in SNr after injection of TVA helper virus but fails to label presynaptic input cells in striatum. Scalebars are 250µm (left) and 500µm (right).

bioRxiv preprint doi: https://doi.org/10.1101/2024.08.28.610136; this version posted September 18, 2024. The copyright holder for this preprint (which was not certified by peer review) is the author/funder, who has granted bioRxiv a license to display the preprint in perpetuity. It is made available under aCC-BY 4.0 International license.

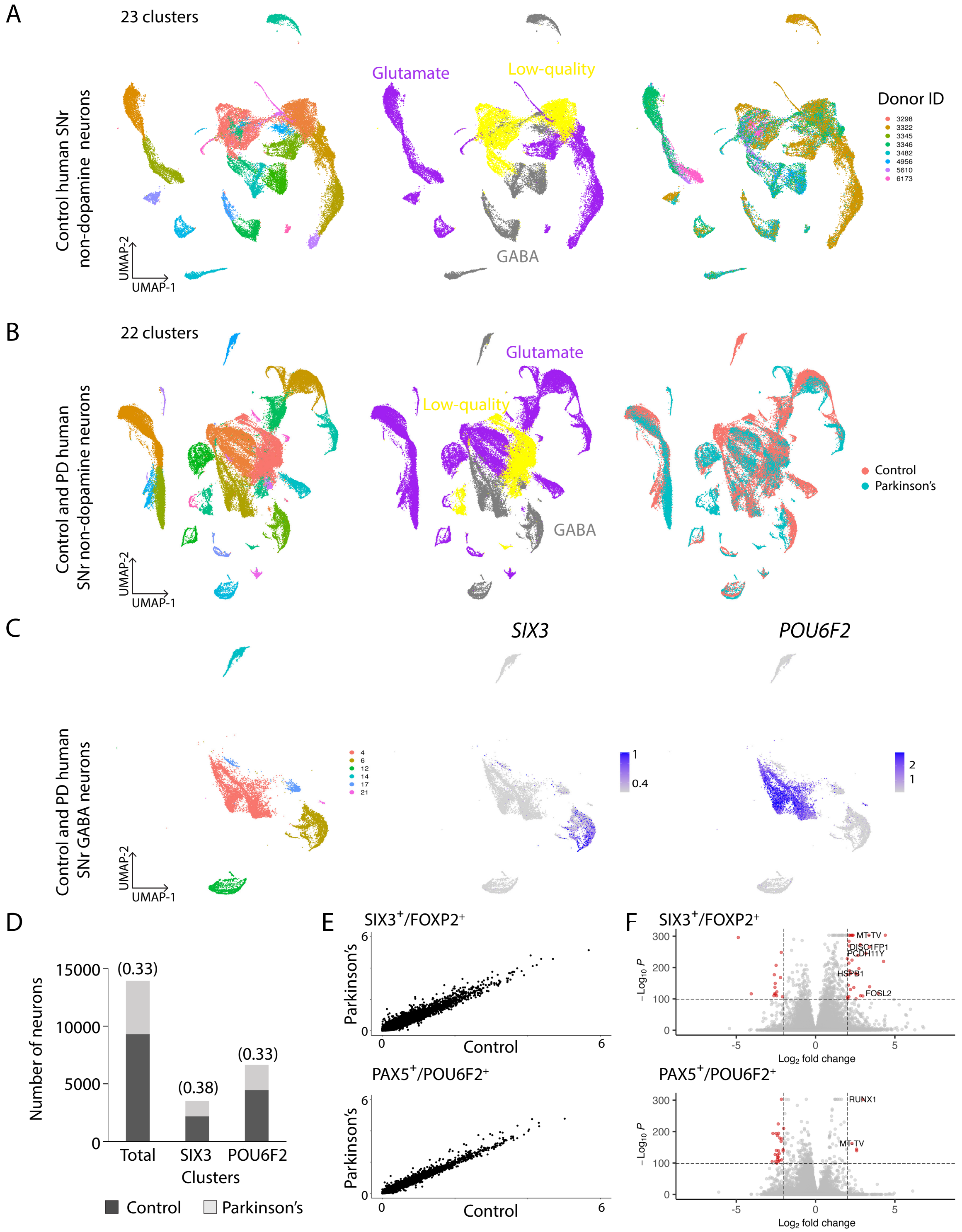

Figure S5. Single-cell transcriptomic profiling of control and Parkinson's human SNr neurons, related to Figure 5

(A) UMAP representation of 39110 human non-dopaminergic midbrain neurons from 8 control donors, colored by cluster (left), cell type label (middle) and donor ID (right).

(B) UMAP representation of 73889 human non-dopaminergic midbrain neurons from 8 control donors (39110 neurons) and 7 Parkinson's Disease donors (34779 neurons), colored by cluster (left), cell type label (middle) and disease state (left).

(C) UMAP representation of 13910 human GABAergic SNr neurons (9307 control neurons and 4603 PD neurons) subsetted from (B) and feature plots showing relative expression of *SIX3* and *POU6F2*.

(D) Number of neurons in the SIX3⁺/FOXP2⁺ and PAX5⁺/POU6F2⁺ clusters deriving from control or PD donors. Proportion from PD donors shown in parentheses.

(E) Average gene expression of control and PD neurons within SIX3⁺/FOXP2⁺ clusters (top) and PAX5⁺/POU6F2⁺ clusters (bottom).

(F) Volcano plots of genes up- or downregulated in PD compared to control within SIX3⁺/FOXP2⁺ clusters (top) and PAX5⁺/POU6F2⁺ clusters (bottom). Genes shown in red have fold change > 2 and p < 10e-100.

bioRxiv preprint doi: https://doi.org/10.1101/2024.08.28.610136; this version posted September 18, 2024. The copyright holder for this preprint (which was not certified by peer review) is the author/funder, who has granted bioRxiv a license to display the preprint in perpetuity. It is made available under aCC-BY 4.0 International license.

Table S1. Select SNr target brains regions and their acronyms, related to Figure 3

PPN	Pedunculopontine nucleus
ZI	Zona incerta
PAG	Periaqueductal gray
DR	Dorsal nucleus raphe
CUN	Cuneiform nucleus
RN	Red nucleus
SCig	Superior colliculus, motor related, intermediate gray layer
SCiw	Superior colliculus, motor related, intermediate white layer
SCdg	Superior colliculus, motor related, deep gray layer
ICe	Inferior colliculus, external nucleus
ICc	Inferior colliculus, central nucleus
ICd	Inferior colliculus, dorsal nucleus
PRNr	Pontine reticular nucleus
PRNc	Pontine reticular nucleus, caudal part
GRN	Gigantocellular reticular nucleus
PARN	Parvicellular reticular nucleus
IRN	Intermediate reticular nucleus
VAL	Ventral anteriorlateral complex of the thalamus
VM	Ventral medial nucleus of the thalamus
MD	Mediodorsal nucleus of thalamus
CM	Central medial nucleus of the thalamus
PF	Parafascicular nucleus
RT	Reticular nucleus of the thalamus
PIL	Posterior intralaminar thalamic nucleus
PoT	Posterior triangular thalamic nucleus
MGv	Medial geniculate complex, ventral part
MGm	Medial geniculate complex, medial part
MGd	Medial geniculate complex, dorsal part

bioRxiv preprint doi: https://doi.org/10.1101/2024.08.28.610136; this version posted September 18, 2024. The copyright holder for this preprint (which was not certified by peer review) is the author/funder, who has granted bioRxiv a license to display the preprint in perpetuity. It is made available under aCC-BY 4.0 International license.

Table S2. Striatum subdomains and their acronyms, related to Figure 4

CPre	Caudoputamen- rostral extreme
CPr.m	Caudoputamen- rostral, medial
CPr.imd	Caudoputamen- rostral, intermediate, dorsal
CPr.imv	Caudoputamen- rostral, intermediate, ventral
CPr.l.ls	Caudoputamen- rostral, lateral, lateral strip
CPr.l.vm	Caudoputamen- rostra,l lateral, ventromedial
CPi.dm.dl	Caudoputamen- intermediate, dorsomedial, dorsolateral
CPi.dm.im	Caudoputamen- intermediate, dorsomedial, intermedial
CPi.dm.cd	Caudoputamen- intermediate, dorsomedial, central dorsal
CPi.dm.dt	Caudoputamen- intermediate, dorsomedial, dorsal tip
CPi.vm.vm	Caudoputamen- intermediate, ventromedial, ventromedial
CPi.vm.v	Caudoputamen- intermediate, ventromedial, ventral
CPi.vm.cvm	Caudoputamen- intermediate, ventromedial, central ventromedial
CPi.dl.d	Caudoputamen- intermediate, dorsolateral, dorsal
CPi.dl.imd	Caudoputamen- intermediate, dorsolateral, intermedial dorsal
CPi.vl.imv	Caudoputamen- intermediate, ventrolateral, intermedial ventral
CPi.vl.v	Caudoputamen- intermediate, ventrolateral, ventral
CPi.vl.vt	Caudoputamen- intermediate, ventrolateral, ventral tip
CPi.vl.cvl	Caudoputamen- intermediate, ventrolateral, central ventrolateral
CPc.d.dm	Caudoputamen- caudal, dorsal, dorsomedial
CPc.d.dl	Caudoputamen- caudal, dorsal, dorsolateral
CPc.d.vm	Caudoputamen- caudal, dorsal, ventromedial
CPc.i.d	Caudoputamen- caudal, intermediate, dorsal
CPc.i.vm	Caudoputamen- caudal, intermediate, ventromedial
CPc.i.vl	Caudoputamen- caudal, intermediate, ventrolateral
CPc.v	Caudoputamen- caudal, ventral
CPce	Caudoputamen- caudal extreme
AcbC	Accumbens nucleus, core region
AcbSh	Accumbens nucleus, shell region
LAcbSh	Lateral accumbens, shell region
